# Reliability Analysis of Complex PCB Assemblies Under Temperature Cycling and Random Vibration

**DOI:** 10.3390/mi16020212

**Published:** 2025-02-13

**Authors:** Wenchao Tian, Feiyang Li, Mang He, Haoyue Ji, Si Chen

**Affiliations:** 1 School of Electro-Mechanical Engineering, Xidian University, Xi’an 710000, China; lifeiyang_233@163.com (F.L.); cameljihy@126.com (H.J.); 2 State Key Laboratory of Electromechanical Integrated Manufacturing of High-Performance Electronic Equipments, Xidian University, Xi’an 710000, China; 3 China Zhenhua Group Yongguang Electronic Co., Ltd., Guiyang 550000, China; 17775200926@163.com; 4 The 58th Research Institute of China Electronics Technology Group Corporation, Wuxi 214062, China; 5 The Fifth Electronics Research Institute of Ministry of Industry and Information Technology, Guangzhou 510000, China; chensiceprei@yeah.net

**Keywords:** temperature cycling, random vibration, thermal-force coupling, reliability

## Abstract

This paper examined the reliability of complex PCB assemblies under random vibration and temperature cycling, which are two primary causes of assembly failure. A combination of finite element simulation and environmental testing was employed to investigate the effects of different reinforcement methods and solder joint morphology on assembly reliability. The linear accumulation of damage was utilized to predict assembly failure, and the predicted failure damage was compared with the damage extracted post-testing to validate the simulation analysis. The results indicate that SAC305 solder exhibits greater strength than Sn63Pb37 solder in withstanding temperature cycling fatigue, yet is weaker than Sn63Pb37 solder in withstanding random vibration fatigue. When the solder is Sn63Pb37, the temperature cycling life of the assembly with the bottom filled and the corners fixed is reduced by 92.3% and 99.3%, respectively, compared to the unreinforced method, while the random vibration life is enhanced by 84 times and 3.9 times, respectively. An increase in pad diameter is advantageous for improving the random vibration life of the assembly, but results in a decrease in the temperature cycling life. When the lower pad diameter ranges from 0.35 mm to 0.55 mm, the assembly temperature cycling life decreases by 28.83%, 82.03%, 90.66%, and 91.22% with the increase of the lower pad diameter, and the random vibration life improves by 4.8 times, 9.5 times, 20.4 times, and 33.6 times, respectively. The predicted locations of vulnerable solder joints for the assembly are consistent with the experimental results, and the failure prediction accuracy of the assembly is 88.89%.

## 1. Introduction

With the swift advancement of the electronics industry, electronic devices are trending toward higher density, miniaturization, and enhanced reliability. Consequently, the packaging of electronic equipment and the reliability of soldering have become subject to increasingly stringent demands. Ball grid array (BGA) packaging stands out for its higher packaging density, shorter signal paths, and superior heat dissipation capabilities. The reliability of solder joints is pivotal to the overall reliability of assemblies and assemblies [1,2]. In temperature cycling environments, thermal stresses may occur when different assemblies are combined due to mismatches in the coefficients of thermal expansion between materials. This can lead to thermal fatigue damage or even failure of the solder joints. In a random vibration environment, the deformation of the printed circuit board (PCB) induces significant alternating stress on the solder joints where the assembly is attached to the board, ultimately resulting in brittle fracture and failure of these joints.

Extensive research has been conducted by numerous scholars on the reliability of electronic assemblies under temperature cycling [3,4,5,6,7], random vibration [8,9,10], and thermal coupling [11] conditions. Kanai et al. [12] discovered that rapid thermal cycling induces equal biaxial stress in solder joints, leading to the sequential formation and expansion of cross-shaped cracks. As crack density increases, crack expansion is hindered by inter-crack collisions, resulting in the formation of fatigue crack networks. Gao et al. [13,14] optimized the structural parameters of solder joints using a response surface regression equation for thermal stresses within the joints and a particle swarm optimization algorithm. Their findings indicated that the critical stress point is located at the contact area between the solder joints and the Cu pads, and they proposed an optimal combination of structural dimensions. Li et al. [15] investigated the temperature cycling fatigue characteristics of POP stacked chip assemblies and developed a method to enhance the temperature cycling reliability of such assemblies. They observed that the stress in the solder joints of the top package is significantly lower than that in the bottom package, with the maximum stress occurring in the inner ring of the middle solder joint. Tian et al. [16] examined the thermal behavior and reliability of Through-Silicon Via (TSV) structures in multilayer silicon adapter plates under temperature cycling conditions. They concluded that peak stress occurs at the intersection surface between the TSV and the Redistribution Layer (RDL), identifying this as the most vulnerable location within the TSV structure.

Other researchers have ventured into predicting solder joint life based on comprehensive reliability studies [17,18]. An et al. [19] conducted an in-depth investigation into the reliability of plastic-encapsulated ball grid array (BGA) assemblies through a series of tests including thermal cycling, random vibration, and coupled thermal–vibration testing. They meticulously analyzed the failure modes of solder joints under these conditions. Their findings revealed that the reliability of assemblies under coupled thermal–vibration conditions is markedly inferior compared to when subjected to individual stressors. Specifically, failures under thermal cycling are predominantly characterized by ductile fracture of the solder, while those under random vibration are largely attributed to brittle intermetallic compounds. Under coupled thermal–vibration conditions, both failure modes coexist. Tian et al. [20] utilized Anand’s intrinsic equation to forecast the thermal fatigue life of ceramic package structures for Film Bulk Acoustic Resonator (FBAR) filters. They explored the impact of solder spillage on assembly temperature reliability and concluded that complete solder spillage results in a higher thermal fatigue life compared to partial spillage. Huang et al. [21] introduced a novel ontological model, building upon existing frameworks, to predict the thermo-mechanical fatigue life of solder joints by calculating the cumulative creep strain of the solder layer. This model, when compared against experimental data, demonstrated enhanced accuracy in predicting solder joint life. Cui et al. [22,23] examined the reliability of airborne electronic assemblies under temperature cycling, random vibration, and composite loading scenarios. Their research indicated that the fatigue life under composite loading is significantly reduced compared to single-factor testing, with failures primarily occurring at the interface between solder joints and copper pads. They successfully predicted the fatigue life under diverse environmental conditions.

In the present study, various life prediction models and unified intrinsic models are employed to forecast the lifespan of solder joints. Additionally, the impact of different solder joint morphologies on assembly reliability is examined, thereby offering insights into enhancing the reliability of electronic assemblies. Despite these efforts, there remains a notable gap in research concerning thermal–vibration coupling and integrated simulation-experimental studies. In this paper, we simulate random vibration and temperature cycling conditions and predict the life of printed board assemblies using finite element analysis software (Ansys 2019 R2). Subsequently, the damage data obtained from these simulations are linearly superimposed and juxtaposed with the actual crack damage observed in solder joints post-testing. This comparison serves to validate the simulation analysis and provides recommendations for improving the assembly’s reliability. The findings of this thesis are an important guide for the design and reliability improvement of electronic devices, especially in high-density, high-performance electronic devices, to improve the reliability of components by optimizing solder joint design and reinforcement methods. Meanwhile, this paper can provide data support and supplement the reliability test standards for electronic devices, and promote the improvement and development of industry standards.

## 2. Materials and Methods

### 2.1. Geometry Model and Relevant Parameters

**Figure 1:**
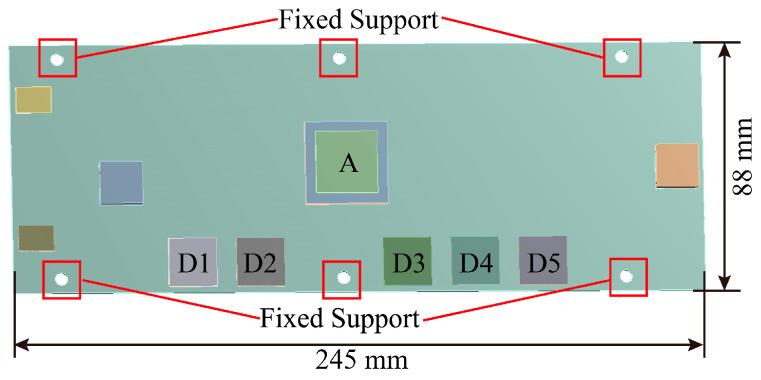
Assembly distribution and modeling.

The layout of the complex PCB examined in this study is depicted in Figure 1. A total of 11 assemblies are distributed across this PCB assembly, with assemblies A and D1–D5 being the focal points of this investigation. The PCB in question is a 10-layer board, measuring 245 mm in length, 88 mm in width, and 2 mm in thickness. Six bolt holes are present on the board, which are utilized for securing and constraining the assembly.

Within the PCB assembly, Assembly A employs the bottom filler adhesive UF3808 to reinforce the bottom solder joints. For Assemblies D1 through D5, the size of the solder joints beneath the pad (adjacent to the PCB-side pad) increases sequentially, resulting in variations in solder joint morphology. The specific dimensions of these assembly parameters are detailed in Table 1.

Assembly A is the main function chip on the PCB board with high speed and high performance, which adopts the FCBGA (Flip Chip Ball Grid Array) process, by inverting the chip and utilizing the ball solder as the electrical connection point to connect the chip directly to the package substrate. Assemblies D1–D5 need to integrate more functions into a limited space, and therefore adopt the FBGA (Fine Pitch Ball Grid Array) process; through the cable technology, the chip’s gold wires are connected to the pads on the package substrate, and the chip is connected face up.

**Figure 2:**
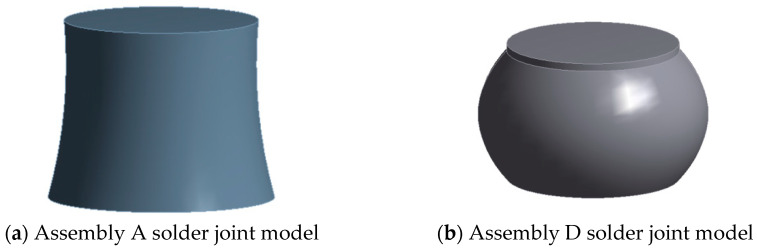
Assembly solder joint model.

The solder joint model is established according to the actual assembly of solder joints, and the structure of the solder joint model is shown in Figure 2.

**Figure 3:**
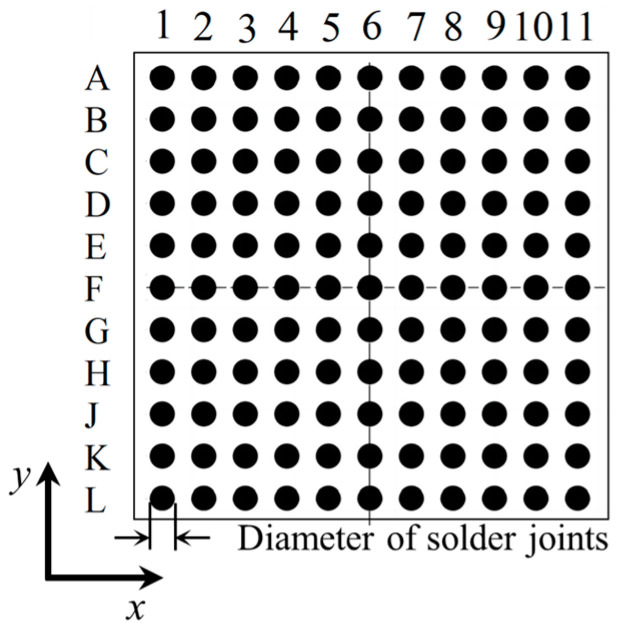
Schematic diagram of solder joint numbering.

To facilitate the description of the solder joints, the assembly solder joints are numerically indexed as illustrated in Figure 3. The top left corner of the solder joint array is designated as A1. The numerical sequence increases progressively in the positive direction of the x-axis, while the alphabetical sequence advances sequentially in the negative direction of the y-axis.

The relevant material parameters in the simulation calculations are shown in Table 2.

The modulus of elasticity of SAC305 and Sn63Pb37 solder varies with temperature as shown in Table 3.

The melting points of the two solder alloys, SAC305 and Pb63Sn37, utilized in the printed board assembly are 217 °C and 183 °C, respectively. The temperature range for the temperature cycling simulation in this study spans from −55 °C to 100 °C. Given that the maximum absolute temperature of the thermal load exceeds half of the solder’s melting point, it is imperative to account for the creep characteristics of the solder. In this context, the Anand viscoplastic unified model is selected to characterize the viscoplastic behavior of the solder. Table 4 presents the nine pertinent parameters of the Anand viscoplastic unified model for both solder alloys.

### 2.2. Meshing and Calculation Conditions

#### 2.2.1. Meshing

The quality of meshing in finite element simulations significantly influences the convergence of the results, thereby impacting both the accuracy and computational efficiency of the simulations. Consequently, selecting an appropriate meshing method is of paramount importance. Generally, a finer mesh with a greater number of elements leads to more accurate simulation outcomes. However, an increase in mesh density can result in a substantial rise in computational time and resource requirements. In this study, a hybrid meshing approach utilizing both hexahedral and triangular elements was employed. To assess mesh independence, four different mesh densities were established and analyzed, as detailed in Table 5.

**Figure 4:**
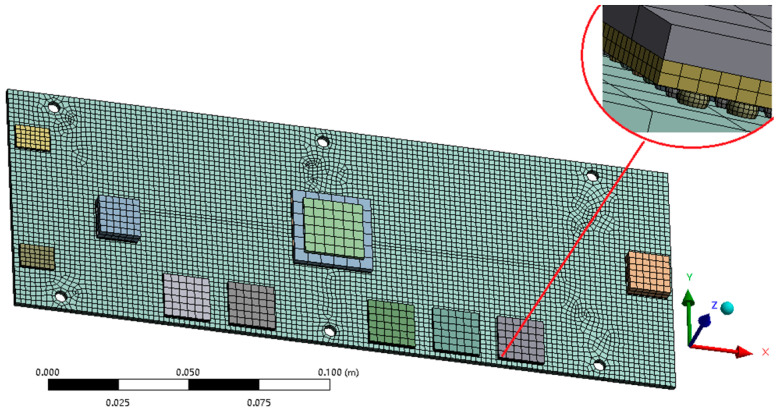
PCB assembly meshing.

Table 5 presents a comparison of the minimum temperature across the entire plate and the intrinsic frequency of a specific section at 453 s under various meshing configurations. As the mesh is refined and its quality enhanced, both the minimum temperature and the intrinsic frequency exhibit a trend toward stabilization. This stabilization indicates that the influence of meshing on the simulation results has been mitigated, ensuring that the calculations are robust against mesh-induced variability. To optimize computational time and resource utilization in this study, the “III” mesh division method was chosen. This method is specifically illustrated in Figure 4, where all contact surfaces are configured to maintain bound contact conditions.

#### 2.2.2. Load Conditions

**Figure 5:**
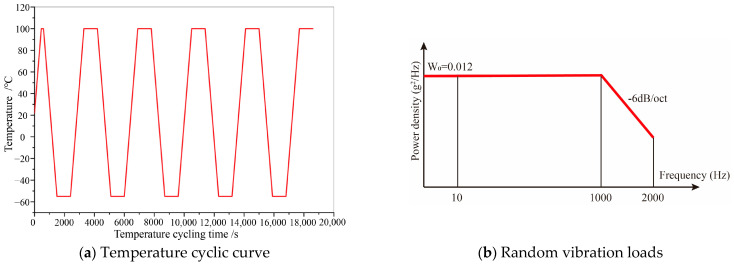
Load conditions.

Based on the actual operating scenario of this PCB, the temperature loading range was set from −55 °C to 100 °C, with an initial temperature of 22 °C in this study. The dwell times at both high and low temperatures were 900 s, and the ramp times were also 900 s, resulting in a total cycle time of 3600 s. Research has indicated that the mechanical properties of welded joints tend to stabilize after five cycles of alternating load [24,25]. Consequently, only five cycles of temperature cyclic loading were applied in the simulation presented in this paper, as depicted in Figure 5a. The temperature load was ramped from 22 °C to 100 °C over 453 s, and the cyclic temperature load was initiated after maintaining the high temperature for an additional 147 s. The rate of both temperature increase and decrease was maintained at 10.33 °C/min.

Random vibration loads were applied to six bolt holes, all subjected to broadband random spectra with a frequency range spanning from 10 Hz to 2000 Hz. The vibration protocol involved sequential one-hour vibrations along the x, y, and z axial directions, constituting a three-hour cycle. Based on the actual operating scenario of this PCB, the frequency range and amplitude for the functional vibration were specified as: 10~1000 Hz at 0.012 g^2^/Hz; and 1000~2000 Hz with a decay rate of −6 dB/oct. The random vibration loads utilized in this simulation are illustrated in Figure 5b.

### 2.3. Numerical Equations

The numerical equations involved in the numerical calculation process are shown below.

Thermal stress equation:

(1) $$\left\{\varepsilon \right\}=\left\{\varepsilon^{el}\right\}+\left\{\varepsilon^{th}\right\}=\Delta T{\left[\begin{array}{cccccc} a_{x} & a_{y} & a_{z} & 0 & 0 & 0 \end{array}\right]}^{T}+{\left[E\right]}^{-1}\left[\sigma \right]$$

where $\left\{\varepsilon \right\}$ is the total strain vector, $\left\{\varepsilon^{th}\right\}$ is the thermal strain vector, $\left\{\varepsilon^{el}\right\}$ is the elastic strain vector, $a_{x}$, $a_{y}$, and $a_{z}$ are the coefficients of thermal expansion of the material in the *x*, *y*, and *z* directions respectively, ΔT is the temperature change, $\left[E\right]$ is the stress–strain matrix of the material, and $\left[\sigma \right]$ is the stress matrix.

Thermodynamic coupling equation:

(2) $$\left[\begin{array}{cc} 0 & 0\\ 0 & C_{T} \end{array}\right]\left[\begin{array}{c} \overset{•}{U}\\ \Delta \overset{•}{T} \end{array}\right]+\left[\begin{array}{cc} K_{u} & K_{uT}\\ 0 & K_{T} \end{array}\right]\left[\begin{array}{c} U\\ \Delta T \end{array}\right]=\left[\begin{array}{c} L_{S}+L_{V}\\ P \end{array}\right]$$

where $C_{T}$ is the specific heat capacity matrix, $\overset{•}{U}$ is the displacement matrix of the nodes, $K_{T}$ is the thermal conductivity matrix, $K_{u}$ is the structural stiffness matrix, P is the temperature loading matrix, $L_{S}$ is the area loading, and $L_{V}$ is the volume loading.

Anand viscoplastic unified model:

(3) σ=cs

where c is a material parameter, c < 1, and s is an internal variable.

(4) $$c=\frac{1}{\xi }\sinh^{-1}\left[\frac{\overset{•}{\varepsilon_{p}}}{A}\left(\exp{\left(\frac{Q}{TR}\right)}^{m}\right)\right]$$

where ξ is the stress multiplier, $\overset{•}{\varepsilon_{p}}$ is the inelastic strain rate, A is the pre-exponential coefficient factor, Q is the activation energy, T is the absolute temperature, and m is the strain rate sensitivity index.

(5) $$\overset{•}{s}=\left[h_{0}{\left|1-\frac{s}{s^{*}}\right|}^{a}\cdot sign\left(1-\frac{s}{s^{*}}\right)\right]\cdot {\overset{•}{\varepsilon }}_{p}$$

where $h_{0}$ is the strain hardening constant, a is the strain hardening index, $s^{*}$ is the initial value of deformation impedance, $\hat{s}$ is the coefficient of saturation value of deformation impedance, and n is the strain rate sensitivity index of saturation value.

Random vibration equation:

(6) $$\left[M\right]\left\{\ddot{x}\left(t\right)\right\}+\left[K\right]\left\{x\left(t\right)\right\}=\left\{0\right\}$$

where $\left[M\right]$ is the mass matrix, $\ddot{x}\left(t\right)$ is the node acceleration column vector, $\left[K\right]$ is the stiffness matrix, and $x\left(t\right)$ is the node displacement column vector.

Temperature cycling fatigue life prediction equation:

(7) $$N_{f}=\frac{1}{2}{\left(\frac{\Delta \gamma_{p}}{2\varepsilon_{f}}\right)}^{\frac{1}{c}}$$

(8) $$c=-0.442-6\times 10^{-4}T_{avg}+1.74\times 10^{-2}\times \ln\left(1+f\right)$$

where $N_{f}$ is the number of fatigue life cycles, $\Delta \gamma_{p}$ is the shear plastic strain range, c is the modified fatigue toughness index, f is the cycle frequency, $T_{avg}$ is the average cycle temperature, and the empirical value of the fatigue toughness index $\varepsilon_{f}$ is 0.325.

Random vibration fatigue life prediction equation:

(9) $$\Delta \varepsilon_{t}=\Delta \varepsilon_{el}=3.5\frac{\sigma_{u}}{E}N_{f}^{-0.12}$$

where $\sigma_{u}$ is the tensile strength of the material.

Random vibration damage superimposed equations:

(10) $$D=\frac{n_{1}}{N_{1}}+\frac{n_{2}}{N_{2}}+\frac{n_{3}}{N_{3}}$$

where $N_{1}$, $N_{2}$, and $N_{3}$ are the number of fatigue cycles to failure at 1*σ*, 2*σ*, and 3*σ* strain levels, which can be calculated from Equation (9); $n_{1}$, $n_{2}$, and $n_{3}$ are the number of fatigue cycles at 1*σ*, 2*σ*, and 3*σ* strain levels, respectively, and the equations are as follows.

(11) $$n_{1}=0.683N_{0}^{+}T\left(3600\;s/h\right)$$

(12) $$n_{2}=0.271N_{0}^{+}T\left(3600\;s/h\right)$$

(13) $$n_{3}=0.0433N_{0}^{+}T\left(3600\;s/h\right)$$

where $N_{0}^{+}$ is the average number of passes through the zero axis with a positive slope per unit time, calculated as follows:

(14) $$N_{0}^{+}=\frac{1}{2\pi }{\left[\frac{\frac{\pi p_{1}f_{1}Q_{1}}{2\Omega_{1}^{2}}+\frac{\pi p_{2}f_{2}Q_{2}}{2\Omega_{2}^{2}}+\cdots }{\frac{\pi p_{1}f_{1}Q_{1}}{2\Omega_{1}^{4}}+\frac{\pi p_{2}f_{2}Q_{2}}{2\Omega_{2}^{4}}+\cdots }\right]}^{\frac{1}{2}}$$

where $p_{i}$ is the value of power spectral density corresponding to the intrinsic frequency, $f_{i}$ is the intrinsic frequency, $Q_{i}$ is the transmission rate, and $\Omega_{i}$ is the intrinsic circular frequency, which is calculated as follows:

(15) $$Q_{i}=\sqrt{f_{i}}$$

(16) $$\Omega_{i}=2\pi f_{i}$$

## 3. Results and Discussion

### 3.1. PCB Assembly Temperature Cycling Analysis

#### 3.1.1. Assembly Stress/Strain Analysis

**Figure 6:**
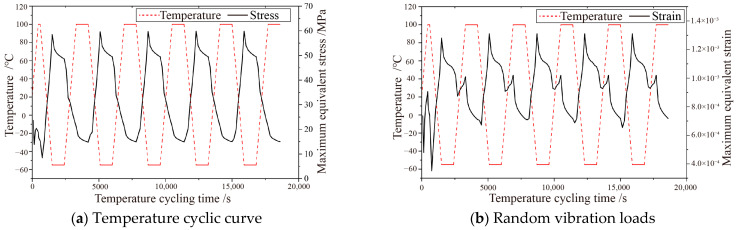
Maximum equivalent stress and strain of soldered joints.

The stress and strain versus time curves for the extracted solder joint array are presented in Figure 6. The stress curves of the solder joints indicate a consistent trend in stress variation across each cycle. During the heating phase, the solder joints exhibit a softening phenomenon, with the maximum equivalent stress gradually decreasing. In the high-temperature holding stage, there is a slight decrease in the maximum equivalent stress of the solder joint, reaching its minimum value at the end of this stage. During the cooling phase, the solder joint begins to harden progressively, with the maximum equivalent stress increasing accordingly. At the end of the cooling phase, the maximum equivalent stress attains its peak value. In the low-temperature holding phase, the accumulated plastic deformation of the joint is gradually released, leading to a minor decrease in the maximum equivalent stress.

From Figure 6, it is evident that the trend of the equivalent strain of the solder joints mirrors that of the maximum equivalent stress, with both the maximum equivalent stress and strain of the solder joint arrays peaking at 15,900 s. The temperature cycle life of the solder joints is forecasted using the temperature and frequency-modified Coffin–Manson equation. The predicted lifespan for each assembly is detailed in Table 6.

#### 3.1.2. Effect of Reinforcement Type and Solder Joint Morphology on Assembly Temperature Cycling Reliability

Building upon the preceding reliability assessments, this investigation delves deeper into the impact of various reinforcement techniques applied to the assembly, as well as the morphological alterations of solder joints resulting from variations in the diameter of the underlying pads, on the temperature cycling reliability of the assembly. This study aims to elucidate how these factors influence the thermal–mechanical behavior and longevity of solder joints under cyclic thermal stress, thereby providing insights for enhancing the overall reliability of electronic assemblies.

(1) Temperature cycling analysis of different reinforcement methods

In practical applications, it has been observed that the choice of reinforcement method significantly influences the reliability of Assembly A. Consequently, this study examines the effect of different reinforcement strategies on the reliability of Assembly A. Four distinct reinforcement schemes have been devised, as detailed in Table 7.

**Figure 7:**
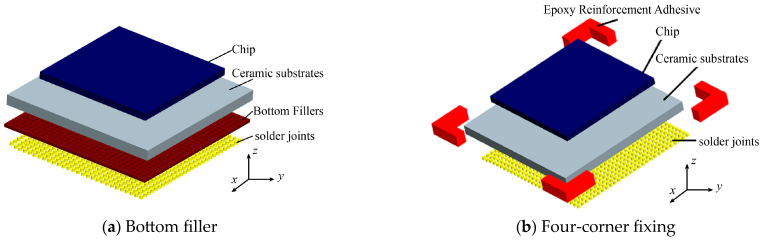
Exploded view of Assembly A model.

During the analysis, modifications are made exclusively to the reinforcement of Assembly A, while the remainder of the assembly model and the analytical procedures remain consistent with the simulation process previously described. Figure 7 presents an exploded view of the model for the bottom-filled Assembly A and the four corners of the fixed Assembly A.

**Figure 8:**
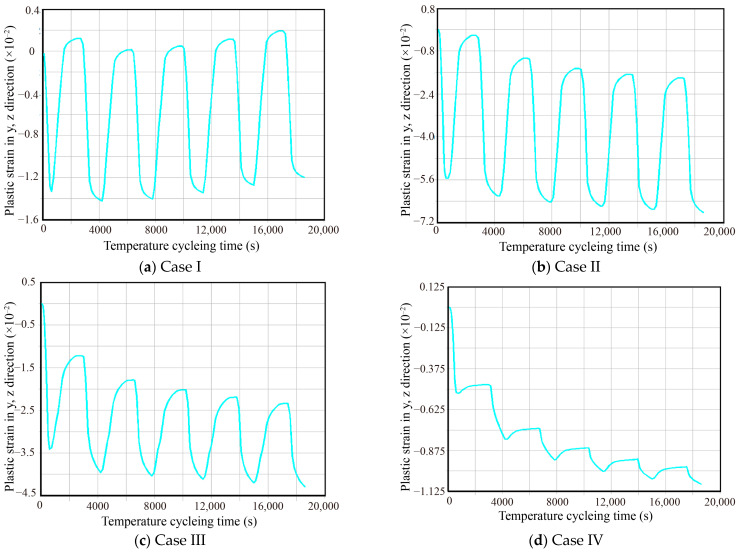
Plastic strain in y, z direction for vulnerable solder joints with different reinforcement types.

The plastic strain curves in each direction of the hazardous location were extracted. After comparison, it is found that the hazardous node of the welded joint has the largest amplitude of plastic strain in the y, z direction. The plastic strain curves in the direction of the maximum plastic strain amplitude (y, z) corresponding to the critical solder joints for each reinforcement method were extracted. These curves are presented in Figure 8.

**Figure 9:**
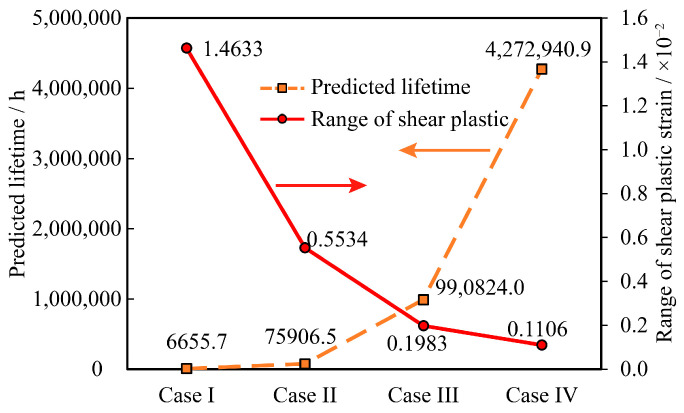
Comparison of temperature cycling results of different reinforcement methods.

The shear plastic strain ranges $\Delta \gamma_{p}$ in the fifth cycle of the plastic strain curves corresponding to the four types of reinforcement in Figure 8 were extracted (14.633 × 10^−3^ for Case1, 5.534 × 10^−3^ for Case2, 1.983 × 10^−3^ for Case3, and 1.106 × 10^−3^ for Case4), and substituting them into Equation (7), we calculated the temperature of Assembly A at four-corner fixation conditions. The predicted cycle life of Assembly A under the four-corner fixed condition is 6655.7 h, 75,906.5 h, 990,824.0 h, and 4,272,940.9 h, respectively. The results of these predictions are illustrated in Figure 9.

As depicted in Figure 9, the fatigue life of the SAC305 solder assembly is 4,772,940.9 h, and that of the Sn63Pb37 solder assembly is 990,824.0 h in the absence of reinforcement. This indicates that the SAC305 solder exhibits significantly greater resistance to temperature cycling fatigue compared to Sn63Pb37 solder, a finding that aligns with conclusions drawn in prior literature. Furthermore, when using Sn63Pb37 solder, the temperature cycling life of the assembly with bottom filler and the assembly with four corners fixation decreased by 92.3% and 99.3%, respectively, relative to the unreinforced condition. This results in a substantial reduction in the temperature cycling life of Assembly A. Additionally, due to the significant mismatch in the coefficients of thermal expansion between the bottom filler and the solder joints, the temperature cycling life of the bottom-filled solder joints is lower than that of the four-corner fixed method.

(2) Reliability Analysis of Temperature Cycling of Assembly D Solder Joint Patterns

**Figure 10:**
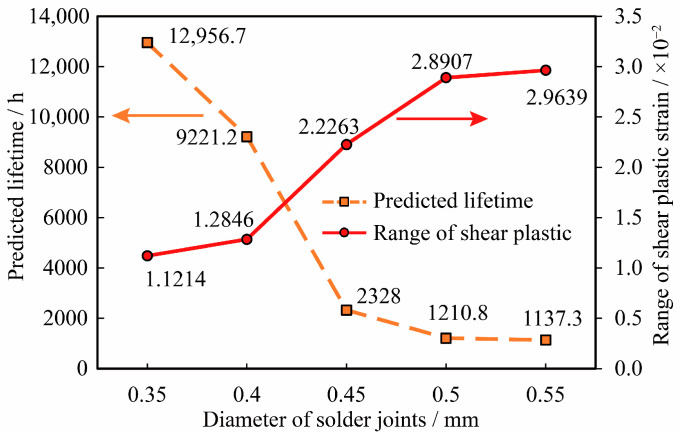
Comparison of temperature cycling results for different pad diameters.

In practical operational scenarios, it has been observed that the morphology of solder joints significantly influences the reliability of Assemblies D1 through D5. Consequently, this paper focuses on investigating the impact of solder joint morphology on the reliability of these assemblies. The simulation calculates the temperature cycling life of the assemblies with varying diameters of the lower pads. The results of these calculations are presented in Figure 10.

From Figure 10, it is evident that when the diameter of the lower pad of the solder joint is within the range of 0.35 mm to 0.55 mm, an increase in the diameter of the lower pad results in a decrease in the temperature cycling life of the assembly. Specifically, compared to a lower pad diameter of 0.35 mm, the temperature cycling life for lower pad diameters of 0.40 mm, 0.45 mm, 0.50 mm, and 0.55 mm is reduced by 28.83%, 82.03%, 90.66%, and 91.22%, respectively. Furthermore, after the lower pad diameter reaches 0.50 mm, the predicted decline in life tends to level off, indicating a stabilization in the temperature cycling life.

### 3.2. Random Vibration Analysis of PCB Assemblies

#### 3.2.1. Modal Analysis

**Figure 11:**
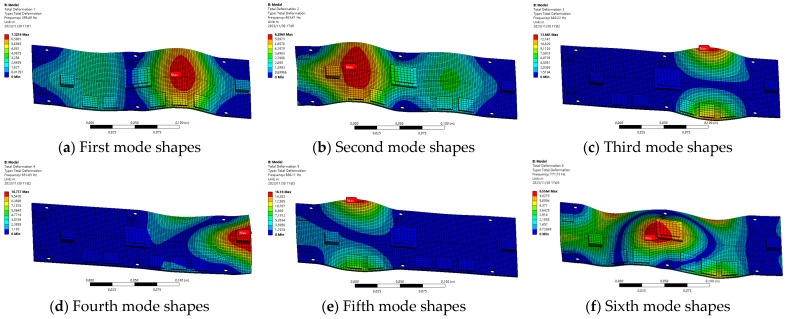
Cloud diagram of the first six orders of modal shapes of PCB assembly.

The modal analysis serves as the foundation for random vibration simulation analysis. Through modal analysis, the modal frequencies and vibration patterns of complex PCB assemblies are obtained. By examining the modal shapes, the deformation of the assembly and the PCB board under random vibration loading conditions can be inferred. The first six orders of modal vibration patterns obtained from the modal analysis are presented in Figure 11.

As illustrated in Figure 11, the maximum amplitude of the first six modal vibration patterns is observed on the printed board. The directions of these modal vibration amplitudes are primarily within the x–y plane, with bending deformations occurring predominantly along the z-direction. Notably, the amplitude near the constrained positions is relatively small, whereas it increases significantly away from these constrained areas. Based on the aforementioned analysis, it can be concluded that the primary damage to the printed board assembly stems from vibrational loads acting in the z-direction. Therefore, a judicious selection of the assembly’s layout location can enhance the assembly’s reliability under random vibration conditions.

#### 3.2.2. Effect of Reinforcement Method and Solder Joint Morphology on Random Vibration Reliability of the Assembly

To optimize the reliability of assemblies in a random vibration environment, this paper compares the random vibration reliability of Assembly A in different reinforcement methods and Assembly D in different solder joint patterns.

(a) Random vibration simulation analysis of Assembly A with different reinforcement methods.

**Figure 12:**
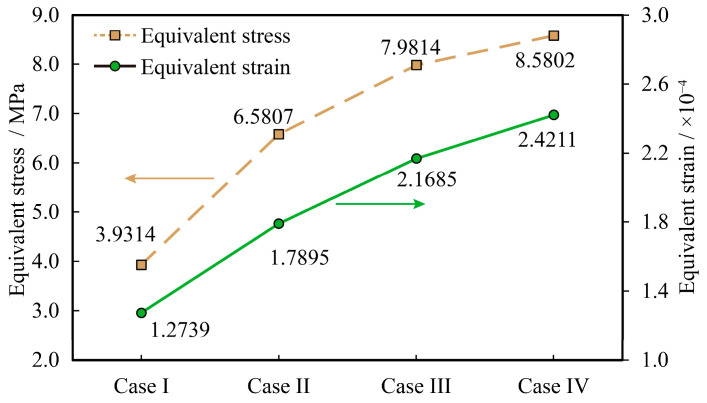
Stress–strain values of vulnerable solder joints in z-direction with the same reinforcement method for Assembly A.

The reinforcement methods for Assembly A are identical to those established in the temperature cycling study, as detailed in Table 7. Modal analysis reveals that the predominant damage to the printed circuit board assembly originates from vibrational loads in the z-direction. Consequently, it is imperative to concentrate on monitoring the stress–strain values of the susceptible solder joints in the z-direction for each reinforcement method. The results depicting the z-direction stress–strain characteristics of the vulnerable solder joints of Assembly A, subjected to the same reinforcement method, are presented in Figure 12.

**Figure 13:**
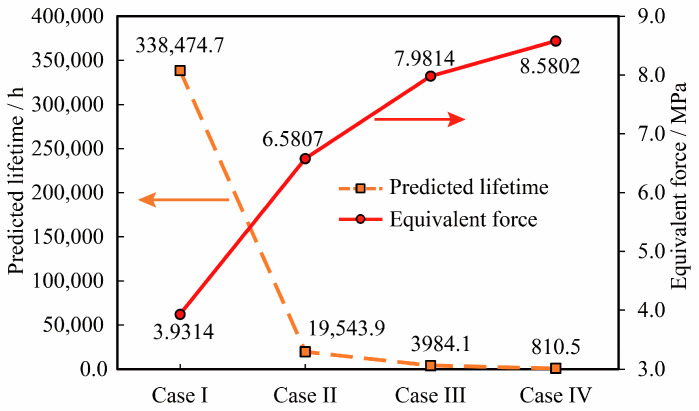
Comparison of random vibration life prediction of Assembly A with different reinforcement methods.

The damage and random vibration predicted life were calculated by Manson’s empirical formula for high weak fatigue, and Figure 13 shows a comparison of the stress values and random vibration life of vulnerable weld joints with different reinforcement methods for Assembly A.

A comparative analysis of random vibration simulation results for various reinforcement methods of Assembly A yields the following findings:

(1) In the absence of reinforcement, the assembly life for SAC305 solder is 810.5 h, whereas, for Sn63Pb37 solder, it is 3984.1 h. The Sn63Pb37 solder demonstrates a superior capacity to endure random vibration fatigue compared to SAC305 solder, a finding that corroborates conclusions drawn in prior literature [26].
(2) For assemblies utilizing Sn63Pb37 solder, the life of the four-corner fixed assembly is 19,543.9 h, and that of the bottom-filled assembly is 338,474.47 h. Relative to the unreinforced method, the random vibration life is enhanced by factors of 3.9 and 84, respectively. Both bottom-filling and corner-fixing significantly extend the random vibration lifetime of Assembly A, with these reinforcement methods proving markedly more effective than the non-reinforced approach. Reinforcement serves to mitigate solder joint deformation and reduce stress within the joints, thereby augmenting the random vibration life of the solder joints.
(3) Random vibration reliability analysis of Assembly D solder joint pattern

**Figure 14:**
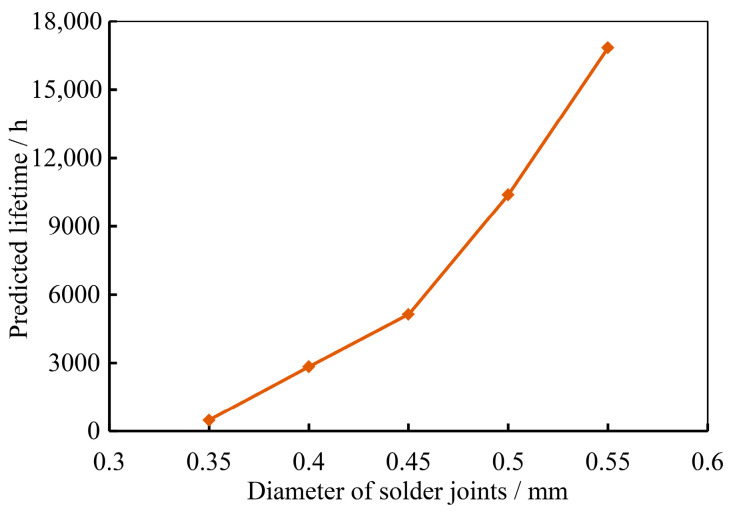
Curve of influence of solder joint morphology on random vibration life of assembly.

To further explore the impact of solder joint morphology changes, induced by variations in the lower pad size, on the reliability of assemblies under random vibration, comparative simulation calculations were conducted for Assemblies D1 through D5. The trend of the random vibration lifetime of Assembly D with different solder joint morphologies, as a function of the lower pad size, is illustrated in Figure 14.

The impact of changes in solder joint morphology, induced by variations in the lower pad diameter, on the temperature cycling life of the assembly, was analyzed. The results indicate that when the lower pad diameter of the solder joint is within the range of 0.35 mm to 0.55 mm, an increase in pad diameter is beneficial for enhancing the random vibration life of the assembly. Specifically, compared to a pad diameter of 0.35 mm, the random vibration life for pad diameters of 0.40 mm, 0.45 mm, 0.50 mm, and 0.55 mm is improved by 4.8 times, 9.5 times, 20.4 times, and 33.6 times, respectively. The rate of increase in life gradually accelerates with the enlargement of the lower pad diameter. In scenarios where increasing the pad size is not feasible, the random vibration reliability of solder joints can be enhanced in practical engineering applications by optimizing the layout, shape, and soldering process of the pads.

The results of the vibration analysis regarding the morphology of the solder joints are opposite to the results of the temperature cycling analysis. Therefore, it is necessary to focus on the actual working conditions when designing and selecting different welding joint diameters according to different working conditions. For equipment that operates for a long time in an environment with high temperatures and frequent temperature changes, such as servers, automotive electronics, aerospace equipment, etc., the temperature cycle life is a key factor to ensure the long-term stable operation of the equipment, and therefore a smaller diameter of the welded joints is required; for the equipment that is used in a mobile, vibration-frequent environment, such as mobile equipment, electronic devices in transportation, military and aerospace equipment, etc., the vibration life is a key factor to ensure the structural integrity and functional stability of the equipment. For equipment used in mobile, vibration-intensive environments, such as mobile equipment, electronic equipment in transportation, military, and aerospace equipment, vibration life is a key factor in ensuring the structural integrity and functional stability of the equipment, thus requiring smaller solder joint diameters.

### 3.3. Analysis of Experimental Results

#### 3.3.1. Metallographic Analysis of Vulnerable Solder Joints

**Figure 15:**
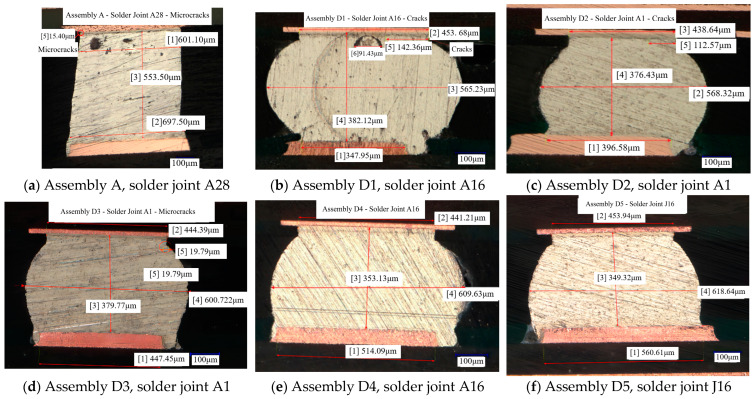
Metallography of vulnerable solder joints of assemblies.

The assembly solder joints were polished and subsequently placed under a metallurgical microscope for detailed observation. By continuously adjusting the magnification, fine cracks, voids, and conditions related to weld quality within the solder joints were meticulously examined. Additionally, dimensional information of the solder joints and the lengths of the cracks were accurately measured. Metallographic images of the solder joints exhibiting the longest cracks, as well as those predicted to be most vulnerable in each assembly, were extracted and are presented in Figure 15.

As depicted in Figure 15, the thickness of the intermetallic compound (IMC) layer within the solder joints falls within acceptable limits. However, the presence of voids in the solder joints poses a significant concern for the reliability of these joints. Notably, cracked solder joints are predominantly observed at the periphery of the array, with cracks predominantly occurring in solder joints adjacent to the assembly’s side.

In this study, a criterion for solder joint failure was established based on the extent of crack propagation post-environmental testing. Specifically, solder joints were deemed to have failed when the crack length surpassed 25% of the total joint length. Conversely, if the crack length remained within 25% of the total length, the presence of cracks was not considered indicative of failure. Among the six assemblies under investigation, two solder joints were identified as having failed. Additionally, two solder joints exhibited cracks but did not meet the failure criterion. One solder joint showed no signs of cracking, as detailed in Table 8.

#### 3.3.2. Comparative Analysis of Test and Simulation Results

Damage data derived from the temperature cycling and random vibration simulation analyses of the welded joints were extracted. These damages are calculated by linear superposition with reference to Equation (10) to predict the failure of the welded joint, following the criterion that failure occurs when the cumulative damage value reaches or exceeds 1. To validate the simulation method’s efficacy in predicting welded joint failure, the predicted damage was compared with the actual cracked welded joints obtained from the validation tests. The damage values from 100 h of temperature cycling simulation and 54 h of random vibration simulation were aggregated to compute the predicted total damage, the results of which are presented in Table 9.

Table 10 presents the predicted total damage and crack control for the vulnerable solder joints of the assemblies. For assembly D2, the solder joint crack length exceeds 25% of the total length. In contrast, the solder joint crack lengths for Assemblies A, D3, D4, and D5 do not surpass 25% and align with the simulation-predicted outcomes, thereby accurately forecasting the cracking conditions of these solder joints. Notably, Assembly D1 exhibits a crack-to-total length ratio of 31.38%, yet the simulated total damage value is 0.822. This discrepancy indicates that the simulation’s total damage prediction does not correspond with the actual crack condition of the solder joint, highlighting a divergence between the simulation results and empirical observations.

This investigation encompassed a total of nine assemblies, wherein the predicted damage levels for eight assemblies were found to be in concordance with the observed experimental cracking conditions. Notably, the cracking pattern of Assembly D1 diverged from the predictions made through simulation. The resultant damage prediction accuracy stood at 88.89%, a figure that corroborates the initially hypothesized outcomes. Moreover, the predictive identification of locations prone to solder joint vulnerabilities within the assemblies was validated by the test results. Table 11 provides a detailed account of the damage characteristics for select solder joints within Assembly D1, highlighting those instances where discrepancies between prediction and observation were noted.

In this study, the failure prediction for all solder joints, except for solder joint A16, was found to be consistent with the experimental outcomes. Specifically, the prediction accuracy for solder joints located along the edge of Assembly D1 was exceptionally high at 98.5%. The simulation successfully predicted the locations of vulnerable solder joints within the assembly, aligning closely with the empirical test results. Notably, the accuracy of failure prediction for solder joints along the edges of the assembly was high, with the sole exception being Assembly D1. These findings substantiate the efficacy of the simulation methodology in analyzing solder joint failures, thereby validating its application in predictive failure analysis.

## 4. Conclusions

In this study, the reliability of printed board assemblies under random vibration and temperature cycling conditions is examined through a combination of finite element simulation and environmental testing. The impact of various reinforcement techniques and solder joint configurations on the assemblies’ reliability under these conditions is analyzed. The linear accumulation of damage is employed to forecast failures following temperature cycling and random vibration tests. The predicted failure damage of the assemblies is juxtaposed with the damage extracted post-testing to affirm the validity of the simulation analysis and to offer recommendations for enhancing assembly reliability. The findings indicate that SAC305 solder demonstrates superior resistance to temperature cycling fatigue compared to Sn63Pb37 solder, yet exhibits inferior performance in withstanding random vibration fatigue. For assemblies soldered with Sn63Pb37, the temperature cycling life is significantly reduced—by 92.3% and 99.3% respectively—when the bottom is filled and the corners are fixed, as opposed to the unreinforced method. However, the random vibration life is notably enhanced, increasing by 84 times and 3.9 times respectively under these reinforcement conditions. An increase in pad diameter is found to be advantageous for improving the assembly’s resistance to random vibration, albeit at the cost of reduced temperature cycling life. Specifically, when the lower pad diameter ranges from 0.35 mm to 0.55 mm, the assembly’s temperature cycling life decreases progressively by 28.83%, 82.03%, 90.66%, and 91.22% with increasing pad diameter, while the random vibration life correspondingly improves by 4.8 times, 9.5 times, 20.4 times, and 33.6 times. The predicted locations of vulnerable solder joints within the assemblies align with experimental outcomes, and the accuracy of failure prediction for the assemblies is determined to be 88.89%. These results underscore the efficacy of the simulation approach in predicting solder joint failures and provide valuable insights for optimizing assembly design and reliability.

## Figures and Tables

**Table 1:** Assembly-related dimensions and information.

Assembly	Package Type	Length (mm)	Width (mm)	Height (mm)	Number of Solder Joints	Solder Joint Distance (mm)
A	FCBGA	29	29	3.75	784	1.00
D1	FBGA	17	17	1.58	256	1.00
D2	FBGA	17	17	1.57	256	1.00
D3	FBGA	17	17	1.56	256	1.00
D4	FBGA	17	17	1.55	256	1.00
D5	FBGA	17	17	1.54	256	1.00

**Table 2:** Table of material parameters.

Materials	Density (g/cm^3^)	Modulus of Elasticity (GPa)	Poisson’s Ratio	Coefficient of Thermal Expansion (×10^−6^/K)	Thermal Conductivity W/(m·°C)	Specific Heat Capacity J/(kg·°C)
Molded material	1.9	19.425	0.3	13	0.9	1180
BT Substrate	1.7	23.1	0.21	12.4	0.2	920
Cu	8.93	128	0.34	16.5	521.5	356.8
SAC305	7.37	Refer to Table 3	0.35	24.5	50	218
Sn63Pb37	8.4	Refer to Table 3	0.36	24.7	50	183
FR-4	1.859	11	0.28	13.6	0.29	1100
Ceramic substrates	3.87	82	0.33	23	117	924
PCB	2.048	11	0.28	16.5	x, y direction: 29.32	1100
					z-direction: 0.3884	
Bottom fillers	1.16	2.61	0.33	55	0.45	1050
Epoxy reinforcing adhesive	1.56	6.5	0.32	30	0.58	980

**Table 3:** Variation of modulus of elasticity with temperature for SAC305 and Sn63Pb37.

**SAC305**											
Temperature (°C)	−40		25		50			125		200	
Modulus of elasticity (MPa)	45,700		34,300		29,090			16,700		3500	
**Sn63Pb37**											
Temperature (°C)	−80	−65		−55		0	25		65		105
Modulus of elasticity	45,700	34,300		29,090		25,812	30,000		30,629		12,455

**Table 4:** Solder Anand parameters.

	Parameter Type								
	*S*_0_ (MPa)	*Q*/*R* (1/K)	*A* (1/s)	*ξ*	*m*	*h*_0_ (MPa)	*Ŝ* (MPa)	*n*	*α*
SAC305	45.9	8314	5.87 × 10^6^	2	0.0942	9350	58.3	0.015	1.5
Sn63Pb37	12.41	9400	4.0 × 10^6^	1.5	0.303	1378.95	13.79	0.07	1.3

**Table 5:** Mesh irrelevance analysis.

Mesh Division Method	Number of Cells	Number of Nodes	Mesh Quality	Temperature Analysis		Vibration Analysis	
				Minimum Temperature at 453 s (°C)	Error (%)	First Natural Frequency (Hz)	Error (%)
I	209,333	1,149,421	0.75258	98.451	0.710	480.82	20.358
II	240,041	1,319,821	0.7721	98.724	0.435	435.82	9.094
III	382,243	2,109,810	0.82879	99.155	-	399.49	-
IV	501,453	2,728,366	0.86498	99.156	0.001	399.48	0.003

III as basis for error calculation.

**Table 6:** Lifetime prediction results for each assembly of PCB assembly.

Assembly	Vulnerable Solder Joints	Amplitude of Shear Plastic Strain (m/m)	Maximum Strain Direction	Predicted Lifetime (h)
A	A28	29	29	3.75
D1	P11	17	17	1.58
D2	N16	17	17	1.57
D3	A16	17	17	1.56
D4	H16	17	17	1.55
D5	J16	17	17	1.54

**Table 7:** Four different reinforcement types.

Case	Reinforcement Types
I	Sn63Pb37 solder, bottom filler
II	Sn63Pb37 solder, four-corner fixing.
III	Sn63Pb37 solder, unreinforced
IV	SAC305 solder, unreinforced

**Table 8:** Cracks in vulnerable solder joints of assembly.

Assembly	Vulnerable Solder Joints	Solder Joint Crack Length (μm)	Diameter of Solder Joints (μm)	Percentage of Crack Length	Failure
A	A28	15.40	601.10	2.56%	No
D1	A16	142.36	453.68	31.37%	Yes
D2	A1	112.57	438.64	25.66%	Yes
D3	A1	19.79	444.39	4.45%	No
D4	A16	0	441.21	0%	No
D5	J16	0	453.94	0%	No

**Table 9:** Analysis of simulated damage in vulnerable solder joints of assemblies.

Assembly	Vulnerable Solder Joints	Temperature Cycling Lifetime (h)	Temperature Cycling Damage	Random Vibration Lifetime (h)	Random Vibration Damage	Total Damage
A	A28	6655.7	1.502 × 10^−2^	7.046 × 10^7^	7.664 × 10^−7^	0.0150
D1	A16	498,104.2	2.008 × 10^−4^	65.7	0.822	0.822
D2	A1	194,894.2	5.131 × 10^−4^	51.0	1.059	1.059
D3	A1	16,544,422.6	6.044 × 10^−6^	312.0	0.173	0.173
D4	A16	64,199.3	1.558 × 10^−3^	3106.6	1.738 × 10^−2^	0.0189
D5	J16	1137.3	8.79 × 10^−2^	2.994 × 10^8^	1.804 × 10^−7^	0.0879

**Table 10:** Comparison of cracks and damage in vulnerable solder joints of assemblies.

Assembly	Vulnerable Solder Joints	Percentage of Crack Length	Total Damage for Solder Joint Simulation
A	A28	2.56%	0.0150
D1	A16	31.38%	0.822
D2	A1	25.66%	1.059
D3	A1	4.45%	0.173
D4	A16	0	0.0189
D5	J16	0	0.0879

**Table 11:** Comparison of cracks and damage in vulnerable solder joints of Assembly D1.

Vulnerable Solder Joints	Crack Length (μm)	Diameter of Solder Joints (μm)	Percentage of Crack Length	Total Damage for Solder Joint Simulation
A1	27.68	453.69	6.10%	6.396 × 10^−2^
A16	142.36	453.68	31.38%	0.822
T1	101.35	446.35	22.71%	0.651
T16	45.91	452.35	10.14%	0.630

## Data Availability

Data are contained within the article.
